# Social media for cardiac imagers: a review

**DOI:** 10.1093/ehjci/jeae109

**Published:** 2024-04-23

**Authors:** Mark J Schuuring, Shehab Anwer, Steffen E Petersen, Sarah Moharem-Elgamal, Denisa Muraru

**Affiliations:** Amsterdam Cardiovascular Science, University of Amsterdam, Meibergdreef 9, 1105 AZ Amsterdam, the Netherlands; Department of Cardiology, Medisch Spectrum Twente, Koningstraat 1, 7512 KZ Enschede, the Netherlands; Department of Cardiology, University Heart Center, Zurich, Switzerland; William Harvey Research Institute, NIHR Barts Biomedical Research Centre, Queen Mary University London, Charterhouse Square, London EC1M 6BQ, UK; Barts Heart Centre, St Bartholomew’s Hospital, Barts Health NHS Trust, West Smithfield, EC1A 7BE London, UK; Department of Cardiology, Liverpool Heart and Chest Hospital, Liverpool, UK; Department of Medicine and Surgery, University of Milano-Bicocca, Milan, Italy; Department of Cardiology, Istituto Auxologico Italiano, IRCCS, Milan, Italy

**Keywords:** social media, publications, cardiology, cardiac imaging techniques, networking

## Abstract

Cardiac imaging plays a pivotal role in the diagnosis and management of cardiovascular diseases. In the burgeoning landscape of digital technology and social media platforms, it becomes essential for cardiac imagers to know how to effectively increase the visibility and the impact of their activity. With the availability of social sites like X (formerly Twitter), Instagram, and Facebook, cardiac imagers can now reach a wider audience and engage with peers, sharing their findings, insights, and discussions. The integration of persistent identifiers, such as digital object identifiers (DOIs), facilitates traceability and citation of cardiac imaging publications across various digital platforms, further enhancing their discoverability. To maximize visibility, practical advice is provided, including the use of visually engaging infographics and videos, as well as the strategic implementation of relevant hashtags and keywords. These techniques can significantly improve the discoverability of cardiac imaging research through search engine optimization and social media algorithms. Tracking impact and engagement is crucial in the digital age, and this review discusses various metrics and tools to gauge the reach and influence of cardiac imaging publications. This includes traditional citation-based metrics and altmetrics. Moreover, this review underscores the importance of creating and updating professional profiles on social platforms and participating in relevant scientific communities online. The adoption of digital technology, social platforms, and a strategic approach to publication sharing can empower cardiac imaging professionals to enhance the visibility and impact of their research, ultimately advancing the field and improving patient care.

## Graphical Abstract

**Graphical Abstract:**
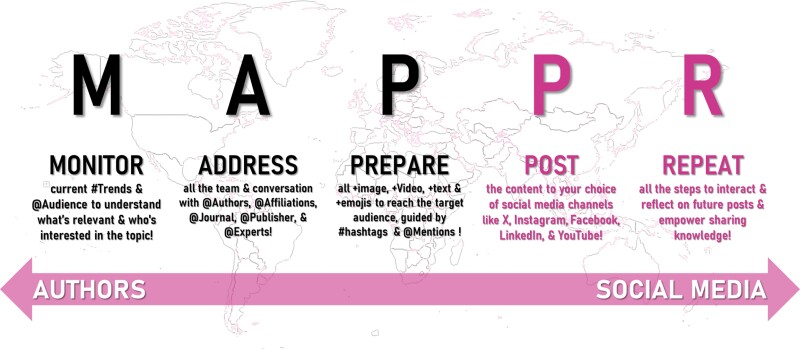
**MAP-PR, a proposed strategy to summarize steps towards an effective post on social media:** A social media post should represent a map of public relation between the author and the target audience on social media, hence, the acronym **MAP-PR**: First the author has to **Monitor** the platform in regard to the topic of interest (e.g. Hashtags #) and identify who is interested (e.g. mentions @) and what are the trending topics, to maximize the impact of the post; second, whether the post discusses a publication or another target, it has to **Acknowledge** those involved in the whole process until its presentation on social media to endorse the team spirit and enhance the discussion from different point of views; the next step is to **Prepare** the post itself, while keeping in mind the target platform (e.g. character count, what to be written in text, and what is better presented in the attached media); as soon as the materials are ready for publication on social media, it is time to **Post** it online and share it among the target audience, when eventually it requires the poster to **Repeat** the aforementioned steps, with a shift of the endpoint on social media: transitioning the focus from releasing and promoting the post-release phase of a social media post to promoting the original post, and allow a fruitful discussion, with the social and personal considerations in mind on the social media platforms.

## Introduction

In the dynamic realm of cardiovascular medicine, the responsibilities within the role of cardiac imagers, who specialize in cardiac imaging from different disciplines including cardiology and radiology, have evolved. Cardiac imagers have a pivotal role in the fields of clinical practice, research, and education. These dimensions require these highly specialized medical professionals to combine clinical expertise, cutting-edge research, and effective knowledge dissemination skills to navigate the intricate and expansive landscape of cardiovascular imaging. First, clinical practice involves the precise diagnosis and risk assessment of cardiac conditions, providing a foundation for a patient-tailored healthcare; second, in research, cardiac imagers are instrumental in studying the novel imaging modalities and techniques, as well as in driving the innovation and scientific progress in the field of cardiac imaging and its reflection on clinical practice; finally, both experience and research would reflect on the education of the newer generations to continue this tuft mission in the different fields of cardiac imaging. Essential for facilitating the timely delivery of dependable scientific evidence, bridging the gap between research and clinical application, and securing optimal patient outcomes across generations, these roles are pivotal in upholding the multifaceted essence of cardiovascular imaging practice within modern healthcare. Effective dissemination of the latest findings is essential for advancing knowledge and improving the quality of care and teaching in the field of cardiovascular medicine.^1^ The emerging availability of social sites, persistent identifiers, and publication-sharing sites has increased the number of options for disseminating research findings.^2^ An organized social media strategy, with a dedicated focus on the social media activity, has become essential to keep up with the role of the cardiac imagers and constitute a bridge between the communities of publisher, author, and the reader. This approach has proven effective to increase engagement with content published in a peer-reviewed imaging journal.^3^

This review aims to provide guidance for cardiac imagers on how to practically improve the utility of social media to improve their publications are easily found, read, used, and cited. Practical advice is given to ensure that every researcher understands how to derive maximum benefit and address potential drawbacks effectively.

### Online visibility

**Figure 1:**
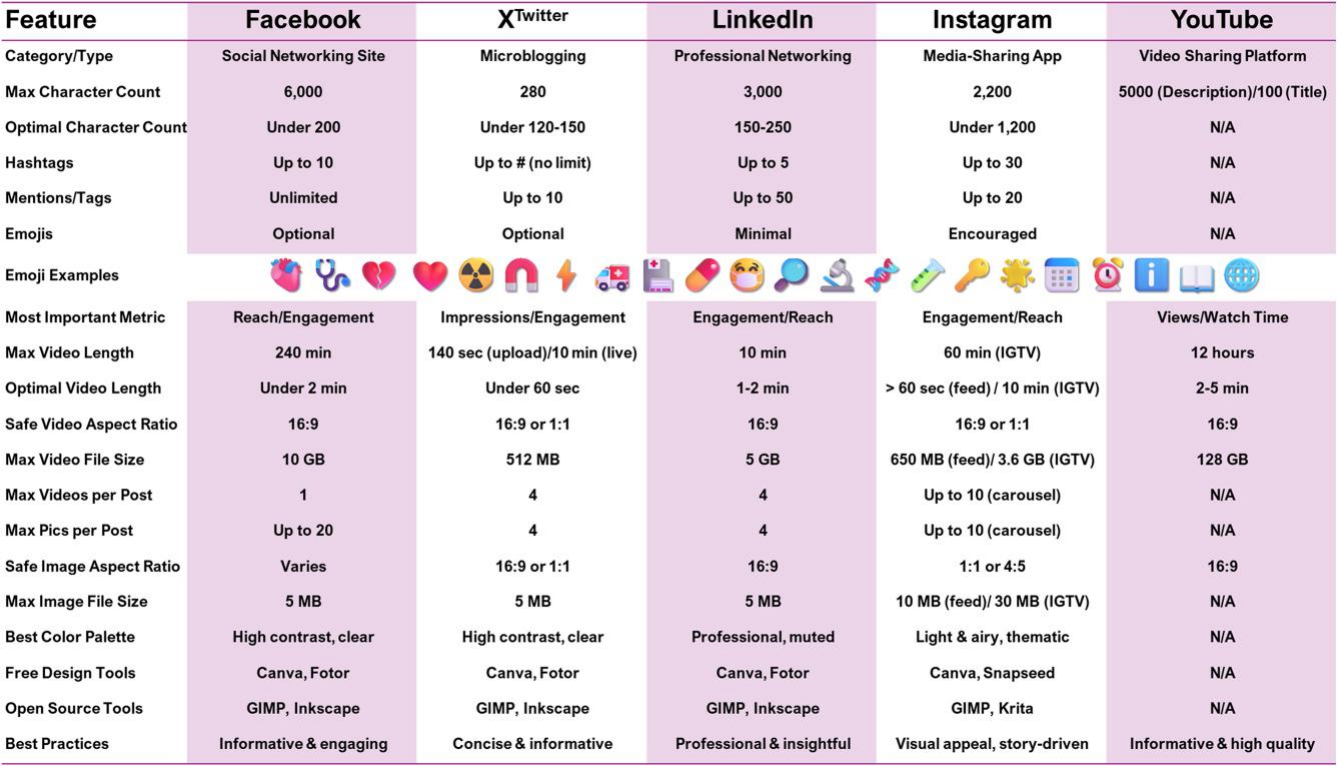
By creating professional profiles and joining relevant scientific communities, cardiac imagers can connect with peers, share their work, and foster collaborations.

By creating professional profiles and joining relevant scientific communities, cardiac imagers can connect with peers, share their work, and foster collaborations (*Figure 1*). One survey reported that the top two reasons for users to create an online account were viewing medical examples and staying informed about questions and answers that were posted.^4^ Cardiac imagers are used to process complex information through imaging and thus translate information into a simple message. Cardiac imagers across the world adopted the use of social sites, persistent identifiers, and publication-sharing sites to share ideas and discuss contemporary issues pertaining to multimodality imaging.^5^ Guidance for sharing patient images and adhering to the General Data Protection Regulation has been described elsewhere.^6^ Increase of dissemination options compels cardiac imagers to reconsider their attitude regarding the most effective communication of their own research findings and educational material to their peers.^7^ Hawkins *et al*. performed a three-arm prospective trial, which was designed using a control group, a basic X (formerly known as Twitter) intervention group (using only the journal’s X account), and an enhanced X intervention group (using the personal X accounts of editorial board members and trainees). Overall, 428 articles published between June 2013 and July 2015 were randomly assigned to the three groups. The enhanced X intervention resulted in a statistically significant increase in both 7- and 30-day X link clicks of the articles compared with the basic X intervention group. Indeed, it is disheartening to invest substantial effort into valuable content that remains unread and undiscovered. For (cardiac) imagers, one study reported on the generation gaps that exist between trainees and faculty, as well as between Generation X and Millennials vs. Baby Boomers, with regard to the use of social media.^8^ The faculty of (cardiac) imagers was more likely than trainees to avoid using social media (30% vs. 9%, *P* < 0.03). Trainees were more likely than faculty to find an electronic case-based curriculum valuable (95% vs. 83%, *P* < 0.05) and were willing to spend more time on cases (*P* < 0.01). Baby Boomers were less interested in joining social media for educational activities than Generation X and Millennials (24% vs. 73%, *P* = 0.0001). Social media has also a growing role in the medical community for applicant decision-making for residents, and the COVID-19 pandemic likely accelerated an inevitable shift in residency programme ‘branding’ and how applicants perceive overall ‘goodness of fit’.^9^ In a survey among 78 residents, 42% reported that social media played a vital role during the application season and 71% reported using social media to learn more about the programme.

Social media can also influence patients’ perspectives of imaging centers.^10^ One study used patient reviews posted on Yelp.com, an online ratings website. A total of 126 outpatient radiology centres from the 46 largest US cities were identified using Yelp.com; 1009 patient reviews comprising 2582 individual comments were evaluated. Overall, 14% of comments were clinician related; 86% pertained to other aspects of service quality. Clinician-related negative comments were more frequent in low-performing centres (mean rating ≤ 2 on 1–5 scale) than high-performing centres (rating ≥ 4) and pertained to imaging equipment (25% vs. 7%), report content (25% vs. 2%), and clinician professionalism (25% vs. 2%) (*P* < 0.010).

### Improve overall online impact

**Figure 2:**
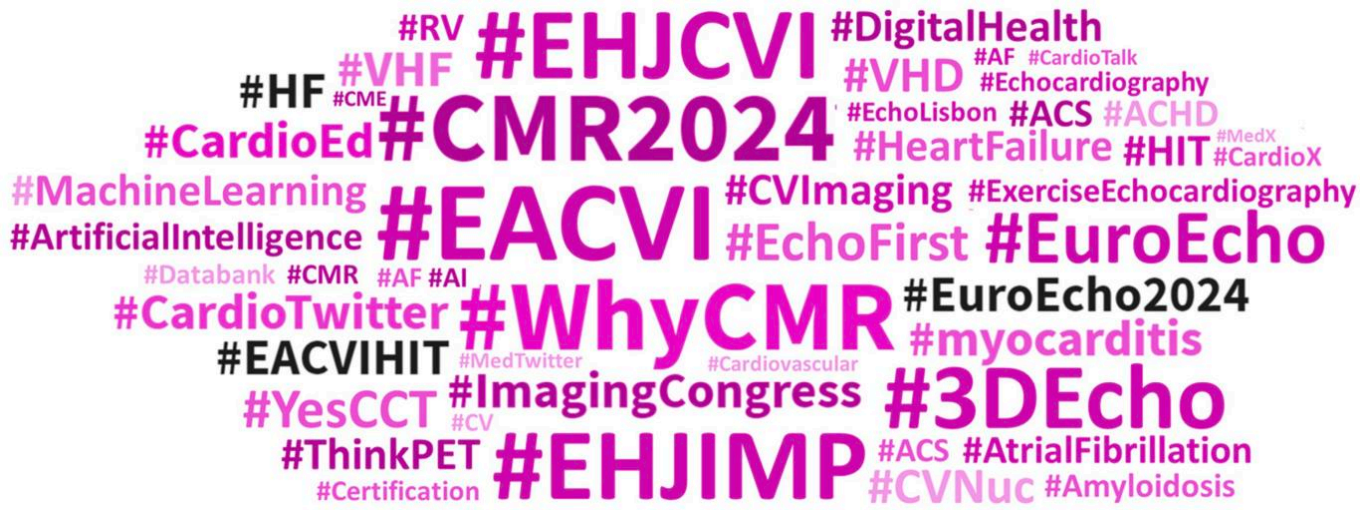
An increasing number of cardiac imagers and cardiac imaging conferences have adopted social media as a means of disseminating highlights.

The delivery of messages through social media channels essentially requires a strategy to maximize its impact.^11^ Leveraging social media allows cardiac imagers to engage with peer cardiac imagers with a broader target audience. The target audience is not exclusively the readers; it includes policymakers, industry professionals, and the general public. An increasing number of cardiac imagers and cardiac imaging conferences have adopted social media as a means of disseminating highlights.^12,13^ Utilize standard hashtags like #ECHOfirst, #yesCCT, and #whyCMR to increase discoverability within the medical community. See *Figure 2*. These hashtags are widely used and recognized globally. Moreover, incorporating visual elements such as emoticons into written messages on social media can enhance their appeal and attract the attention of readers.

Developing powerful messages and accounts is key to a boost in exposure. These should encompass the author’s voice, his/her affiliation, organization, and the publisher’s account. A professional demeanour of the account, focusing on content that reflects relevant information and expertise and maintaining a formal tone and appearance is essential. The best practice is to be authentic and engage with the audience by responding to comments, sharing valuable content, and showing the human side in the background to build trustworthy and meaningful connections. It is advised to include links to other social media platforms in the social media post, which can help connect with more people. Including a link to PubMed when publishing an article makes it easier for readers and researchers to access the research online. Consistent tracking and analysis of the social media metrics is a key to refine the strategy to spot what would work best for the target audience and maximize the impact of the message post and eventually achieve the targets of the post on the social media. This requires the posts to provide concise and engaging summary of the published research and incorporate visually appealing media (such as graphical abstract, example images, or videos), while effectively applying the appropriate hashtags and keywords. This extends after the post release and requires attention to maintain the engagement with the audience, either by responses, invite to participate in the discussions, and expand the vibe on the topic by sharing related content that would build a niche and an active network that promotes the publication and open discussion and enhances its educational impact.

### Platform-specific best practices

Many clinicians use one or multiple social media platforms. A survey among 176 Turkish radiology residents has shown that 10 residents (5.7%) had no social media accounts, while the majority had a Facebook account (139, 79%), followed by 55 (31.3%) on X and 44 (25%) on YouTube.^14^ As fast-growing social media applications worldwide, TikTok and Snapchat are used by a limited number of clinicians but may present important opportunities for contemporary, unique content creation and engagement with non-physician audience.^15,16^*Figure 1* is an overview with key features and options on social media to support the best practice section.

#### X, formerly known as Twitter

For cardiac imagers aiming to achieve maximum impact on X, crafting well-structured tweets that capture attention and foster engagement is essential (*Figure 1*). It is recommended to begin with a clear and concise message regarding the cardiac image or relevant topic, utilizing appropriate medical hashtags to categorize the content and broaden its reach. Visual appeal can be enhanced by including high-quality images or GIFs, which are especially effective in the field of cardiac imaging. To encourage discussions and collaboration, it is common to tag relevant professionals, colleagues, or organizations using ‘@’. The key messages should be kept short within the character limit and should convey valuable insights or educational content. The author of the post should respond promptly to comments and actively engage with the target audience to build a strong online presence in the realm of cardiac imaging.

#### Instagram

For cardiac imagers on Instagram, optimizing content for visual impact and educational value is paramount. Focus on sharing high-quality images and short videos showcasing diverse cardiac imaging modalities, intriguing cases, or procedural insights. Craft informative captions that elucidate the significance of each image or video, providing valuable context for both medical professionals and lay followers. Engage with your audience by encouraging comments, questions, and discussions, fostering a sense of community and knowledge exchange. Collaborate with peers or medical institutions for joint initiatives or shoutouts to broaden your reach. Consistency in posting is key to maintaining engagement and visibility in followers’ feeds, so establish a regular schedule and stick to it. Through these tailored strategies, cardiac imagers can effectively leverage Instagram as a platform for education, professional networking, and public outreach.

#### Facebook

For a significant impact on Facebook, it is essential to structure posts that engage and educate the target audience effectively (*Figure 1*). It is recommended to start with an attention-grabbing introduction to the focus of the post, emphasizing its relevance and value to the target audience through the available relevant channels over Facebook: profiles, pages, and groups. Attachment of high-quality and well-presented media (figures and/or videos) enhances the convey of the key messages, especially when these messages are novel or complex. In addition, a straightforward, conversational tone with minimal use of medical jargon is recommended to deliver the key messages, summary, or highlights and ensure a broader understanding within the diversity of the target audience. Discussion should be encouraged by asking questions or inviting personal experiences related to cardiac imaging, which can foster a sense of community and mutual learning. Finally, one should maintain a consistent posting schedule and interact with comments and messages to build a strong presence and trusted source of information within the realm of cardiac imaging on Facebook.

#### YouTube

Creation of a compelling YouTube video as a cardiac imager to achieve the most impact involves careful structuring. A concise and engaging introduction clearly communicates the video’s purpose and relevance to the target viewers (*Figure 1*). High-quality cardiac images and visuals enhance understanding. The use of concise and simple explanations avoids overwhelming viewers with technical jargon. It is recommended to maintain a steady pace throughout the video, keeping it concise and to the point, while ensuring it’s visually and audibly clear. A call to action can be included at the end, inviting viewers to subscribe, like, share, or comment on the video, which can boost engagement and reach. Regularly engaging with comments and questions fosters a sense of community and trust within the target audience. By combining informative content, engaging visuals, and audience interaction, one can maximize the impact of the published YouTube videos as a cardiac imager.

#### LinkedIn

LinkedIn is increasingly used as a social media platform by clinicians. To maximize the impact of LinkedIn posts for cardiac imagers, it is crucial to create well-structured content that resonates with the available professional network and their affiliated pages or groups (*Figure 1*). The post should start with a compelling introduction, highlighting the significance of the topic discussed, whether it is a new imaging technique, research findings, and/or industry insights. The use of relevant medical and cardiology-related hashtags is a cornerstone to increase the post’s discoverability among the target audience. High-quality images or slides may complement the content and visually engage the connections on LinkedIn. A concise, jargon-free language ensures broad comprehension, and discussion can be stimulated by asking open-ended questions or sharing personal insights related to cardiac imaging. Additionally, one should establish a consistent posting schedule and actively engage with comments and messages to foster a strong professional network and thought leadership in the field of cardiac imaging.

### Considerations

It is important to maximize productivity with minimal concerns about the author’s privacy and the accuracy of the shared information while being mindful of online behaviour and etiquette. For example, oversharing personal information and engaging in online conflicts can have long-lasting consequences on professional image and personal privacy. It is important that all relevant stakeholders are involved.^17^ The potential impact of misinformation and fake news has to be considered, as well as verification of the accuracy of information before they are shared to avoid and even fight the spread of false or harmful content on social media. Ethical considerations and maintaining scientific rigor while using social media are critical topics, especially when dealing with potential patient identifiers, from a straightforward identify exposure up to unique personal identifiers, like body modification (e.g. tattoos or piercings) have to be carefully removed from the media content shared on social media.^18^ Best practices are clear communication of scientific uncertainty, avoiding sensationalism, and citing sources appropriately, essential in promoting accurate information and maintaining trust with the audience. It is recommended to align to activities of the organization, such as European Association of Cardiovascular Imaging, and imaging journals, such as *European Heart Journal – Cardiovascular Imaging* and *European Heart Journal – Imaging Methods and Practice*.

## Conclusion

In conclusion, this review has explored practical advice for cardiac imagers seeking to increase the visibility of their research findings. The findings presented herein highlight social media’s immense potential in enhancing scientific research’s dissemination and impact. First and foremost, it is evident that cardiac imagers must recognize the importance of establishing a solid online presence and engaging actively on social media platforms. Cardiac imagers should adopt a strategic approach to sharing their work on social media. Overall, by incorporating social media into their dissemination strategies, cardiac imagers can extend the reach and impact of their research findings. The recommendations outlined in this review provide practical guidance for cardiac imagers to navigate the realm of social media effectively, ultimately contributing to advancing knowledge and fostering meaningful connections within the scientific community and beyond.

## Data Availability

Data are available upon reasonable request to the corresponding author.
